# Encapsulation of Phenolic Compounds from a Grape Cane Pilot-Plant Extract in Hydroxypropyl Beta-Cyclodextrin and Maltodextrin by Spray Drying

**DOI:** 10.3390/antiox10071130

**Published:** 2021-07-15

**Authors:** Danilo Escobar-Avello, Javier Avendaño-Godoy, Jorge Santos, Julián Lozano-Castellón, Claudia Mardones, Dietrich von Baer, Javiana Luengo, Rosa M. Lamuela-Raventós, Anna Vallverdú-Queralt, Carolina Gómez-Gaete

**Affiliations:** 1 Department of Nutrition, Food Science and Gastronomy XaRTA, Faculty of Pharmacy and Food Sciences, Institute of Nutrition and Food Safety (INSA-UB), University of Barcelona, 08028 Barcelona, Spain; daniescobar01@ub.edu (D.E.-A.); julian.lozano@ub.edu (J.L.-C.); lamuela@ub.edu (R.M.L.-R.); 2 Unidad de Desarrollo Tecnológico, Universidad de Concepción, 4191996 Coronel, Chile; 3 Departamento de Farmacia, Facultad de Farmacia, Universidad de Concepción, 4191996 Concepción, Chile; jaavendano@udec.cl (J.A.-G.); jluengo@udec.cl (J.L.); 4 DEMad, Instituto Politécnico de Viseu, 3504-510 Viseu, Portugal; jsantosu@estgv.ipv.pt; 5 LEPABE—Faculty of Engineering, University of Porto, 4200-465 Porto, Portugal; 6 Consorcio CIBER, M.P. Fisiopatología de la Obesidad y la Nutrición (CIBERObn), Instituto de Salud Carlos III (ISCIII), 28029 Madrid, Spain; 7 Departamento de Análisis Instrumental, Facultad de Farmacia, Universidad de Concepción, 4070386 Concepción, Chile; cmardone@udec.cl (C.M.); dvonbaer@udec.cl (D.v.B.)

**Keywords:** microencapsulation, cyclodextrin, vine shoots, food waste, *Vitis vinifera* L., polyphenols, stilbenoids, mass spectrometry, Fourier Transform Infrared Spectroscopy, Scanning Electron Microscopy

## Abstract

Grape canes, the main byproducts of the viticulture industry, contain high-value bioactive phenolic compounds, whose application is limited by their instability and poorly solubility in water. Encapsulation in cyclodextrins allows these drawbacks to be overcome. In this work, a grape cane pilot-plant extract (GC_PPE_) was encapsulated in hydroxypropyl beta-cyclodextrin (HP-*β*-CD) by a spray-drying technique and the formation of an inclusion complex was confirmed by microscopy and infrared spectroscopy. The phenolic profile of the complex was analyzed by LC-ESI-LTQ-Orbitrap-MS and the encapsulation efficiency of the phenolic compounds was determined. A total of 42 compounds were identified, including stilbenes, flavonoids, and phenolic acids, and a complex of (*epi*)catechin with *β*-CD was detected, confirming the interaction between polyphenols and cyclodextrin. The encapsulation efficiency for the total extract was 80.5 ± 1.1%, with restrytisol showing the highest value (97.0 ± 0.6%) and (*E*)-resveratrol (32.7 ± 2.8%) the lowest value. The antioxidant capacity of the inclusion complex, determined by ORAC-FL, was 5300 ± 472 µmol TE/g DW, which was similar to the value obtained for the unencapsulated extract. This formulation might be used to improve the stability, solubility, and bioavailability of phenolic compounds of the GC_PPE_ for water-soluble food and pharmaceutical applications.

## 1. Introduction

The generation of food and agricultural waste is a growing problem, with negative impacts on the economy, environment, and human health. Therefore, the integral valorization of these wastes by conversion into bioenergy or recovery of chemical compounds for biobased products is a technological challenge for achieving a circular economy. Alternative ways of disposing of food waste include the valorization of byproducts as a source of phenolic compounds used to fortify high-consumption foods or to formulate new functional foods [1].

Grape canes of *V. vinifera* L. produced during pruning are the main byproduct of viticulture, and millions of tons are generated worldwide every year [2,3]. With the aim of developing a circular economy by an integrated biorefinery strategy, grape canes are being investigated as a high-value resource due to their attractive chemical composition and potential industrial applications. These include SO_2_ substitution in wine to improve wine quality [4], usage as a cosmetic ingredient [5] and as a filler in food packaging [4,6], and for the recovery of hemicellulosic oligosaccharides, lignin, and cellulosic substrates [7]. Above all, however, grape canes have been studied for their nutraceutical applications, as they contain high-added-value phenolic compounds with wide-ranging biological properties.

Grape canes contain a complex mixture of phenolic compounds, including phenolic acids (hydroxybenzoic acid and hydroxycinnamic acids), flavonoids (mainly proanthocyanidins, flavonols, flavanonol and flavanones), and stilbenes (monomers, dimers, and oligomers). The most abundant phenolic compounds are proanthocyanidins and stilbene oligomers [8,9]. The phenolic composition of *V. vinifera* plants is genetically determined and strongly influenced by environmental conditions such as water stress [10]. Other determining factors, which particularly affect the stilbenes content, are the cultivar [11], the geographic region of cultivation [12], and the time, temperature, and humidity of storage after pruning [13]. Moreover, the yields of phenolic compounds such as resveratrol and *ε*-viniferin are highly dependent on variables of the extraction methods such as grape cane particle size, the type of solvent, temperature, duration, and the effects of light [14]. Furthermore, the phenolic profile of grape canes can also be altered by the extraction and scale-up process, where oxidation, degradation, or polymerization of proanthocyanidins have been observed, as well as the formation of phenolic aldehydes [2]. Phenolic compounds are known to be unstable and sensitive to high temperature, light, pH, and oxidative and degradative enzymes, which affects the phenolic profile of extracts. It is therefore important to find a strategy to protect phenolic compounds, and preserve their biological activities and properties. Enhancing their bioaccessibility and bioavailability and promoting their transport for absorption by the human body are also of great interest. Accordingly, new approaches, such as encapsulation with cyclodextrins, have been developed to overcome these drawbacks. [15].

Cyclodextrins are highly biocompatible and have been approved by the Food and Drug Administration (FDA) as safe for humans. Several research studies have described the complexation of phenolic compounds with cyclodextrins [15], which provides protection from environmental conditions and improves bioactive shelf-life. Cyclodextrins, which have a truncated cone-shaped structure, possess a hydrophobic interior and a hydrophilic outer surface. Complexation in cyclodextrins improves the water solubility of phenolic compounds, which is otherwise relatively poor. The stability of the complex is maintained via hydrophobic forces, van der Waals interactions and hydrogen bonding [15]. Therefore, encapsulation of the bioactive molecule by cyclodextrin alters the physicochemical properties of both agents. Nevertheless, the effect of cyclodextrins on the profile of phenolic compounds from grape canes after encapsulation needs further study.

In this context, we encapsulated a previously characterized grape cane pilot-plant extract [2] in HP-*β*-CD by a spray-drying technique, using maltodextrin (MD) as a coating material. The physicochemical properties and parameters of the encapsulation process were determined, and the complex formation was verified by scanning electron microscopy (SEM) and Fourier Transform Infrared Spectroscopy with diamond attenuated total reflectance (FTIR-ATR). In addition, the phenolic profile of the inclusion complex was investigated using LC-ESI-LTQ-Orbitrap-MS, and encapsulation efficiency and antioxidant capacity were determined. The microencapsulated extract is envisaged as a functional ingredient of food, cosmetics, biomaterials, and other biobased products.

## 2. Materials and Methods

### 2.1. Chemicals and Reagents

Gallic, 4-hydroxybenzoic, and ellagic acids, catechin, epicatechin, (*E*)-resveratrol, (*E*)-*ε*-viniferin, (*E*)-piceatannol, eriodictyol, taxifolin, quercetin, quercetin-3-*O*-glucoside, and quercetin-3-*O*-glucuronide were purchased from Sigma-Aldrich (St. Louis, MO, USA). Gallic acid and kaempferol-3-*O*-glucoside were acquired from Extrasynthèse (Genay, Auvergne-Rhône-Alpes, France). Isohopeaphenol and hopeaphenol were kindly given by the research group of Prof. Dr. Peter Winterhalter (Institute of Food Chemistry, Technical University Braunschweig, Lower Saxony, Germany). Light exposure was avoided when manipulating the standards.

HPLC-grade acetonitrile, formic acid, ethanol, and water were purchased from Merck (Darmstadt, Hesse, Germany). Ultrapure water was generated by a Milli-Q water purification system Millipore (Bedford, Massachusetts, USA). Potable ethanol (96%) from molasses employed for pilot-plant scale extraction was purchased from Oxiquim S.A. (Coronel, Concepción, Chile).

Hydroxypropyl beta-cyclodextrin (HP-*β*-CD, Kleptose^®^, HP oral grade) was purchased from Roquette Frères (Lestrem, Lillers, France). Maltodextrin (MD) (dextrose equivalent 16.5–19.5) was obtained from Merck KGaA, (Darmstadt, Hesse, Germany).

### 2.2. Pilot-Plant Scale Extraction

Grape cane extraction was performed on a pilot-plant scale (750 L) at 80 °C for 100 min, following our previously reported extraction process [2].

After winter pruning, we collected a total of 500 kg of wet sample grape canes (*V. vinifera* L. cv. Pinot noir) from plants in an organic vineyard at Viña De Neira, located in Ránquil, Itata Valley, Biobio Region, Chile. All samples were cut into 30–50 cm long pieces and stored for three months at room temperature (19 °C ± 5) and 30–70% relative humidity, according to previous reports by Riquelme et al. [16] and Patent [13]. We used 67 kg of dry grape canes from a total sample of 500 kg of wet samples for the pilot-plant extraction, before which grape canes were crushed in a Retsch grinder (model SM) at 300–2000 rpm until particle size was less than 1 cm. After extraction, the ethanol used as a solvent for the extraction was removed and recovered by distillation (absolute pressure 0.05 bar). The liquid extract was collected in a dark container and protected from light.

### 2.3. Preparation of Microcapsules by Spray-Drying

The GC_PPE_ was in a mixed ethanol/water solution (30:70 *v*/*v*). HP-*β*-CD was used to prepare the microcapsules in a proportion of 2.2% *w*/*v* with the extract. MD in a proportion of 10% *w*/*v* was used as the coating material. HP-*β*-CD was slowly added to a beaker containing 200 mL of GC_PPE_ to avoid its agglomeration. The mixture was continuously stirred at room temperature and protected from the light for 24 h, during which the encapsulation took place. Then, MD was added slowly, and the mixture was stirred for a few minutes [17]. The microencapsulated and unencapsulated GC_PPE_ were dried using a Büchi Mini Spray Dryer B-290 (Büchi, Flawil, Switzerland). Inlet temperature was maintained at 130 °C, while the outlet air temperature was 71 °C. Air inlet and airflow were 35–40 m^3^/h and 473 L/h, respectively. The spray dryer was equipped with a nozzle tip diameter of 0.7 mm and a peristaltic pump operated at 6–7 mL/min. The dried powder was collected and stored in an amber airtight container at 4 °C until analysis.

### 2.4. Determination of the Physical Properties of the Microencapsulated Powders

#### 2.4.1. Moisture Content and Total Solids

The moisture content of the samples was calculated from the weight loss after heating the sample to 105 °C for 6 h [18].

#### 2.4.2. Process Yield (PY%)

The yield of the powder process was calculated considering the number of solids introduced into the spray drying system and the powder obtained at the end of the technological process [19]. The results were determined according to Equation (1):

(1) $$\mathrm{PY}\;\left(\%\right)=\frac{\mathrm{Powder}\;\mathrm{after}\;\mathrm{spray}\;\mathrm{dry}}{\mathrm{Solids}\;\mathrm{introduced}\;\mathrm{in}\;\mathrm{the}\;\mathrm{feeding}}\times 100$$

#### 2.4.3. Bulk Density

To determine the bulk density, about 10 g of the spray-dried sample was weighed, placed in a 25 mL graduated test tube, and the occupied volume was recorded [18].

#### 2.4.4. Angle of Repose

The angle of repose was determined using a fixed funnel by the following Equation (2) as in Dadi et al. [18]:

(2) $$\mathrm{Angle}\;\mathrm{of}\;\mathrm{repose}\;\left({}^{\circ}\right)=\tan^{-1}\;\left(H/R\right)$$

where H is the height of the pile and R is its radius at the base.

#### 2.4.5. Size Distribution

The particle size distribution was determined in a particle size analyzer by laser diffraction with a Mastersizer 3000 (Malvern Instruments, Worcestershire, UK). Samples were dispersed in MilliQ water (900 mL) under constant stirring (2300 RPM) using a Hydro EV dispersion unit to achieve a homogeneous suspension. The particle size distribution in the powder (*span*) was calculated using Equation (3):

(3) $$Span=(d90_{\;}-d_{10})/d_{50}$$

where d_90_, d_10_, and d_50_ are the equivalent volume diameters at 90%, 10%, and 50% cumulative volume, respectively [20].

### 2.5. Scanning Electron Microscopy

The microcapsules and GC_PPE_ were analyzed by scanning electron microscopy (SEM) using the JSM 6380 LV system (JEOL Techniques Ltd., Tokyo, Japan). The microscope was operated at 20 kV accelerating voltage. The samples were coated with a gold layer of about 150 Å in thickness, using an Edwards S 150 sputter coater (Agar Scientific, Standsted, UK) [21].

### 2.6. Fourier Transform Infrared Analysis

The FTIR absorption spectra of the individual samples of GC_PPE_, HP-*β*-CD, the inclusion complex (IC) (GC_PPE_+HP-*β*-CD+MD), and the physical mixture were analyzed separately. The physical mixture (PM) (GC_PPE_/HP-*β*-CD/MD) was prepared by accurately weighing HP-*β*-CD (100 mg), GC_PPE_ (100 mg), and MD (20 mg), which were ground in a mortar until the mixture was homogeneous. The resulting physical mixture was immediately analyzed by FTIR-ATR.

The FTIR spectra were recorded using a Bruker Alpha T FTIR (Bruker, Germany) spectrophotometer equipped with a diamond attenuated total reflectance (ATR) unit. The 32 scans were acquired over a spectral range of 4000–500 cm^−1^ with a resolution of 4 cm^−1^ [22,23]. All spectra were acquired and processed using the OPUS 7.0 software.

### 2.7. LC-ESI-LTQ-Orbitrap-MS Analyses

Liquid chromatography (LC) analysis was conducted using an Accela chromatograph (Thermo Scientific, Hemel Hempstead, UK) equipped with a quaternary pump, photodiode array detector, and thermostated autosampler. Chromatographic separation was performed in an Atlantis T3 column 2.1 × 100 mm, 3µm (Waters, Milford, MA, USA). Gradient elution of analytes was performed utilizing H_2_O/0.1% HCOOH (solvent A) and CH_3_CN (solvent B) at a continuous flow rate of 0.350 mL/min, and an injection volume of 5 µL. The following gradient was applied: 0 min, 2% B; 0–2 min, 8% B; 2–12 min, 20% B; 12–13 min, 30% B; 13–14 min, 100% B; 14–17 min, 100% B; 17–18 min, 2% B and the column was equilibrated to the initial conditions for 5 min [24].

The LC equipment was coupled to an LTQ-Orbitrap Velos mass spectrometer (Thermo Scientific, Hemel Hempstead, UK) employed for accurate mass measurements and equipped with an electrospray ionization (ESI) source operating in negative mode. The working parameters were as follows: source voltage, 4 kV; sheath gas, 20 a.u. (arbitrary units); auxiliary gas, 10 a.u.; sweep gas, 2 a.u.; and capillary temperature, 275 °C. Default values were used for most of the other acquisition parameters (FT Automatic gain control target 5·10^5^ for MS mode and 5·10^4^ for MS^n^ mode). Samples were analyzed in FTMS mode with a resolving power of 30,000 (FWHM at *m*/*z* 400) and data-dependent MS/MS events were acquired with a resolving power of 15,000. The most intense ions detected in the FTMS spectrum were chosen for the data-dependent scan. The parent ions were fragmented by high-energy C-trap dissociation by normalized collision energy of 35 V and an activation time of 10 ms. The mass range in FTMS mode was from *m*/*z* 100 to 1500. Instrument control and data recovery were conducted using Xcalibur 3.0 software (Thermo Fisher Scientific). The tentative identification of analytes was performed by comparing MS/MS spectra with fragments found in databases and the literature when no standard compound was available [25].

Individual compounds were semi-quantified using pure standards or the most similar compounds. Some analytes, such as glycosylated forms, dimers, or oligomers, were semi-quantified using the aglycone form of the monomer [2].

### 2.8. Encapsulation Efficiency

Encapsulation efficiency was calculated by considering the nonencapsulated compounds present in the GC_PPE_ before the encapsulation process and the encapsulated compounds after the spray drying process. The identification and quantification of phenolic compounds from GC_PPE_ were specified in a previous article [2] (see detail Supplementary Materials Table S1). Extraction of polyphenols from the microcapsules was performed according to the procedure of Robert et al. [26] with minor modifications. Before the analysis, samples were dissolved in highly pure deionized water containing 0.1% *v*/*v* formic acid (1 mg/mL). The GC_PPE_ was centrifuged at 4000 rpm for 5 min at 4 °C. The supernatant was recovered, and the extraction procedure was repeated twice. The supernatants were combined and evaporated under nitrogen flow, and the residue was reconstituted in 0.1% aqueous formic acid (5 mL). The samples were filtered through 0.20 µm PTFE membrane filters (Waters Corporation, Milford, CT, USA) into an amber vial. Subsequently, both samples were analyzed using LC -ESI-LTQ-Orbitrap- MS to determine the individual degree of encapsulation according to the method described above (Section 2.7). We estimated the encapsulation efficiency (EE), according to Radünz et al. [27], based on Equation (4):

(4) $$\mathrm{EE}\;\left(\%\right)=\frac{\mathrm{Phenolic}\;\mathrm{compound}\;\mathrm{of}\;\mathrm{GCPPE}\;-\;\mathrm{Phenolic}\;\mathrm{compound}\;\mathrm{of}\;\mathrm{the}\;\mathrm{capsule}}{\mathrm{Phenolic}\;\mathrm{compound}\;\mathrm{of}\;\mathrm{GCPPE}}\times 100$$

### 2.9. Antioxidant Capacity Assay

The assay of oxygen radical absorbance capacity using fluorescein (ORAC-FL) was carried out according to the method reported by Ou et al. [28]. The calibration curves were prepared with Trolox, and results reported as µmol Trolox equivalents (TE) by grams of dried weight (DW) (TE/g DW). All assays were performed in triplicate and protected from light.

### 2.10. Statistical Analysis

All samples were run at least three times, and results are expressed as means ± standard deviations. A *p*-value < 0.05 was considered statistically significant using Student’s *t*-test with 95% confidence. Statistical analyses were determined using GraphPad Prism 8.0.1 (GraphPad Software, San Diego, CA, USA).

## 3. Results and Discussion

### 3.1. Physical Characterization of the Microencapsulated Powder

Table 1 shows the physicochemical properties and process parameters for the GC_PPE_ and inclusion complex (IC) (GC_PPE_+HP-*β*-CD+MD). The process yield obtained by spray-drying for IC (GC_PPE_+HP-*β*-CD+MD) was 83.8 ± 2.6%, which was two-fold higher than for GC_PPE_ alone (38.4 ± 1.2%). Similarly, the total solids increased 2.6-fold for the microencapsulated formulation. The high yield of powdered microparticles can be attributed to the rapid formation of the drying crust, which prevents the powder from adhering to the drying chamber [29]. Our result constitutes an improvement on the yield (64.5 ± 1.5%) reported by Davidov-Pardo et al. [30], who microencapsulated a grape seed extract using MD. The high values obtained in our work are promising for the development of industrial-scale applications.

The particle size distribution and median particle diameter were smaller in the IC (GC_PPE_+HP-*β*-CD+MD) than in the GC_PPE_ alone; the particle sizes were 10.9 μm and 17.5 μm, respectively. According to the literature, the diameter of spray-dried particles depends on the properties of the material, the drying conditions, the atomization method used, and the concentration and viscosity of the encapsulated material [31]. The *span* values of the IC (GC_PPE_+HP-*β*-CD+MD) and GC_PPE_ were very similar, 6.14 and 6.15, respectively, and were higher than those reported for an aqueous grape skin extract microencapsulated with Arabic gum, polydextrose, and partially hydrolyzed guar gum, which ranged from 1.91 to 5.99 [32]. A lower *span* value is a desirable result, as it indicates a more homogeneous particle size distribution [32].

The bulk density was 0.10 ± 0.01 g/mL and 0.19 ± 0.01 g/mL for the GC_PPE_ and IC (GC_PPE_+HP-*β*-CD+MD), respectively, being lower than the values reported for encapsulated rosemary essential oil (0.25–0.34 g/mL) [31] or soy milk (0.21–0.22 g/mL) [20]. The slightly higher bulk density of the IC (GC_PPE_+HP-*β*-CD+MD) vs. the GC_PPE_ indicates an improved powder flow, as a more densely packed powder reflects weaker forces between the particles [20]. Density is an important factor for the packaging, transportation, and marketing of a microencapsulated product. A dry product with high density can be stored in a smaller container compared to a less dense product [31]. The flowability of the samples was also determined by the angle of repose, which was 34.8° ± 0.5 for the GC_PPE_ and 36.9° ± 1.3 for the IC (GC_PPE_+HP-*β*-CD+MD), showing no statistical difference between them. A similar value was obtained in a study on the microencapsulation of bioactive products from a *Moringa stenopetala* leaf extract using MD, where the angle of repose was 37.26° ± 1.01 [18].

### 3.2. Surface Morphology: SEM Analysis

**Figure 1:**
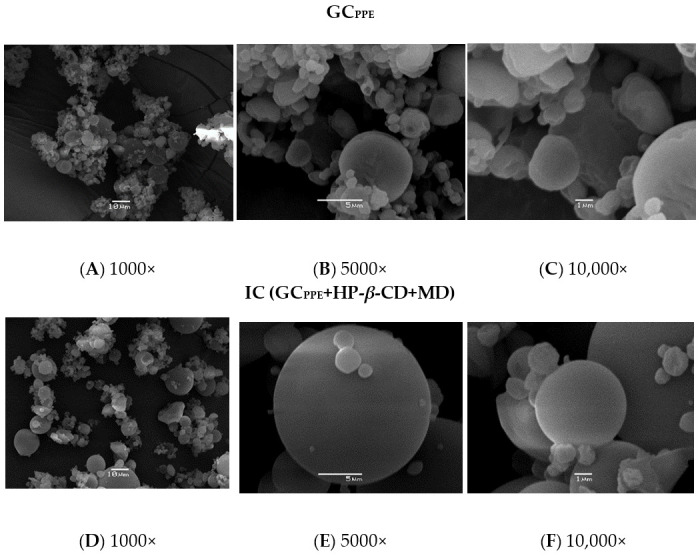
Scanning electron microscopy micrographs of the GC_PPE_ (**A**–**C**) and IC (GC_PPE_+HP-*β*-CD+MD) (**D**–**F**) at different magnifications.

SEM can be used to determine the surface morphology of materials and is recognized as an auxiliary method for monitoring the formation of inclusion complexes. The structure and size of the GC_PPE_ and IC (GC_PPE_+HP-*β*-CD+MD) in the solid state obtained from the spray-drying process were analyzed through microscopy. Microcapsules should preferably have a slightly spherical form and a uniform and smooth cover with minimum fractures and signs of collapse [33]. The SEM micrographs showed that the GC_PPE_ was composed of a mixture of non-spherical particles with irregular surfaces and other larger spherical microparticles (Figure 1A–C). In contrast, the IC (GC_PPE_+HP-*β*-CD+MD) was spherical, and without visible pores on a smooth surface; microparticles of a variable size but with similar morphology were observed together (Figure 1D–F). These significant morphological changes are probably due to a loss of crystallinity of the guest molecule after its inclusion in the cyclodextrin [34]. The SEM results provided evidence for the formation of the IC (GC_PPE_+HP-*β*-CD+MD), which was subsequently supported by mass spectrometry and FTIR analysis.

### 3.3. FTIR Analysis of Spray-Dried Powders

**Figure 2:**
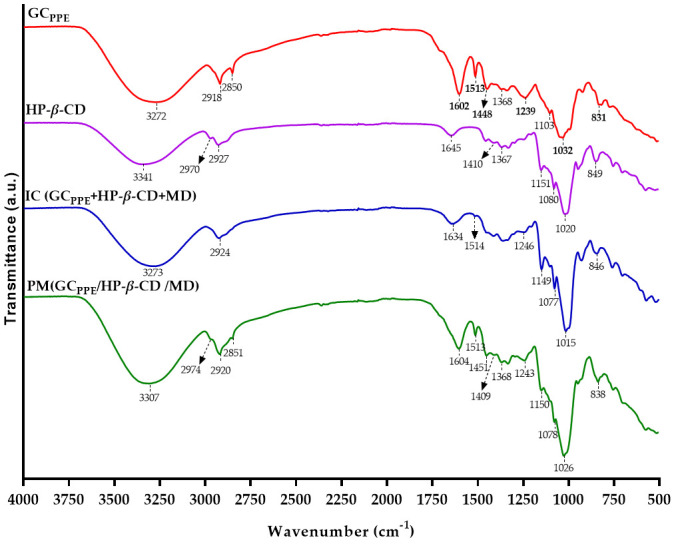
FTIR-ATR spectra of the GC_PPE_ (**red**), HP-*β-*CD (**purple**), IC (GC_PPE_+HP-*β*-CD+MD) (**blue**), and PM (GC_PPE_/HP-*β*-CD/MD) (**green**).

FTIR-ATR is a useful method to detect the formation of inclusion complexes, which are revealed by changes in the FTIR spectra, such as the reduction, disappearance, or shift of absorption bands, due to weak intermolecular interactions [35]. The FTIR spectra of the GC_PPE_, HP-*β*-CD, IC (GC_PPE_+HP-*β*-CD+MD), and physical mixture (PM) (GC_PPE_/HP-*β*-CD/MD) are shown in Figure 2.

The FTIR spectrum of the GC_PPE_ (Figure 2, red) showed specific bands associated with phenolic compounds (in bold). The peaks at 1602 cm^−1^ and 1448 cm^−1^ were owing to the C=C stretching vibration of the phenolic aromatic ring and the C–H bending vibrations of the CH_2_ groups. The peak at 1239 cm^−1^ was assigned to C=O stretching of gallic or ellagic acid components due to the presence of hydrolyzable tannins [36]. The band at 1513 cm^−1^ was due to the C–C benzene skeletal vibrations of stilbenoids. The strong band at 1032 cm^−1^ is attributed to C–O stretching in phenolic compounds [22]. Moreover, the band at 831 cm^−1^ was due to the C-H out-of-plane bending vibrations of aromatic compounds. On the other hand, the signals at 2918 cm^−1^ and 2850 cm^−1^ were related to the CH_2_ asymmetric and symmetric stretch vibration in aliphatic hydrocarbons [37]. Additionally, a peak at 1103 cm^−1^ was observed owing to the C–H in-plane bending vibration [36].

The FTIR spectrum of HP-*β*-CD (Figure 2, purple) showed bands at 3341 cm^−1^ due to O–H stretching vibrations and 2927 cm^−1^ due to C–H stretching vibrations. The peaks at 1645 cm^−1^ correspond to the bending of H–O–H, at 1151 cm^−1^ to C–O vibration, and at 1020 cm^−^^1^ to the C–O–C symmetric stretching vibration. The peak at 849 cm^−1^ was due to an α-type glycosidic bond [38]. The band at 1080 cm^−1^ was ascribed to C–C stretching vibrations, and the peak at 1410 cm^−1^ to C–C–H and O–C–H bending [39]. The presence of the hydroxypropyl group was recognized by a peak at 2970 cm^−1^ corresponding to the antisymmetric vibration of methyl groups. Additionally, a peak was observed at 1367 cm^−1^, which was ascribed to the bending vibration of the methyl group [40].

The spectrum of the IC (GC_PPE_+HP-*β*-CD+MD) (Figure 2, blue) shows that some of the characteristic peaks of the GC_PPE_ and HP-*β*-CD have shifted, decreased, or disappeared. The bands at 1645 cm^−1^ and 1020 cm^−1^ of HP-*β*-CD have shifted to 1634 cm^−1^ and 1015 cm^−1^, respectively, in the IC. Two peaks of the GC_PPE_ at 1239 cm^−1^ and 1513 cm^−1^, ascribed to the presence of hydrolysable tannins and stilbenes, respectively, have shifted to 1246 cm^−1^ and 1514 cm^−1^, showing a sharp reduction in intensity due to the complexation, whereas the peak at 2850 cm^−1^ disappeared. Furthermore, the bands of the GC_PPE_ at 1602 cm^−1^ and 1448 cm^−1^, related to the phenolic aromatic ring, completely disappeared in the IC (GC_PPE_+HP-*β*-CD+MD). These findings may indicate that the phenolic rings became embedded in the HP-*β*-CD cavities.

The FTIR spectrum of the PM (Figure 2, green) showed a simple overlap between the individual components of GC_PPE_, HP-*β*-CD, and MD. Insignificant variations in intensity were detected, indicating a mixture of the three components without interactions between them.

### 3.4. Phenolic Profile of the IC (GC_PPE_+HP-β-CD+MD) by LC-ESI-LTQ-Orbitrap-MS

We performed a targeted analysis of phenolic compounds in the IC (GC_PPE_+HP-*β*-CD+MD) using LC-ESI-LTQ-Orbitrap-MS. Table 2 shows 42 identified compounds with their accurate mass, theoretical mass, retention times (min), molecular formula, error (ppm) between the found mass and the accurate mass of each compound, and the MS/MS fragment ions with their respective intensities used for identification. The identification was further supported by comparisons with mass spectra databases and the literature. In addition, 13 phenolic compounds were identified by comparing the retention times and their masses with pure standards. We have reported the fragmentation patterns of most of these compounds in previous studies using an analytical [8] and pilot-scale extraction [2].

Of the 42 compounds identified in the present work, 22 had been previously detected in both the analytical and pilot-plant extractions [2,8]: gallic acid (*m*/*z* 169.0138, −2.41 ppm), monogalloyl-glucose (*m*/*z* 331.0662, −2.65 ppm), protocatechuic acid-*O*-hexoside (1) (*m*/*z* 315.0718, −1.07 ppm), protocatechuic acid (*m*/*z* 153.0190, −2.16 ppm), protocatechuic acid-*O*-hexoside (2) (*m*/*z* 315.0717, −1.30 ppm), syringic acid hexoside (*m*/*z* 359.0933, 2.21 ppm), catechin (*m*/*z* 289.0722, 1.43 ppm), epicatechin (*m*/*z* 289.0715, −1.00 ppm), restrytisol (A or B) (*m*/*z* 471.1441, −1.86 ppm), taxifolin (*m*/*z* 303.0508, −0.78 ppm), astilbin (*m*/*z* 449.1084, −1.12 ppm), stilbenoid heterodimer (caraphenol B/C) (*m*/*z* 469.1292, −0.06 ppm), eriodictyol-*O*-glucoside (*m*/*z* 449.1089, −0.04 ppm), stilbenoid dimer (*m*/*z* 469.1292, −0.07 ppm), pallidol (*m*/*z* 453.1349, 2.24 ppm), (*E*)-resveratrol (*m*/*z* 227.0715, 0.77 ppm), stilbenoid dimer (resveratrol dimer) (*m*/*z* 453.1351, 1.57 ppm), resvertarol-*O*-hexoside (*m*/*z* 615.1868, −0.62 ppm), eriodictyol (*m*/*z* 287.0558, −0.92 ppm), hopeaphenol (*m*/*z* 905.2607, −2.24 ppm), isohopeaphenol (*m*/*z* 905.2588, 2.15 ppm) and (*E*)-ε-viniferin (*m*/*z* 453.1348, 1.03 ppm). These findings indicate that these compounds are stable through each step of the production process, including microencapsulation.

On the other hand, seven compounds identified in the microencapsulated extract had previously been detected only in the analytical extraction [8]: procyanidin dimer (1) (*m*/*z* 577.1348, −0.69 ppm), procyanidin dimer (2) (*m*/*z* 577.1346, −0.35 ppm), epicatechin gallate (*m*/*z* 441.0821, −1.30 ppm), (*E*)-piceatannol (*m*/*z* 243.0665, 0.79 ppm), viniferin diglycoside (*m*/*z* 777.2397, −0.36 ppm), (*E*)-ω-viniferin (*m*/*z* 453.1346, 0.62 ppm), and stilbenoid tetramer (vitisin A/B/C/D) (*m*/*z* 905.2599, −3.05 ppm). Thus, although these compounds were not detected in the pilot-scale extraction, they were recovered after the microencapsulation process. Similar to our results, the incorporation of *β*-CD enabled an effective and selective recovery of flavan-3-ols [41] and stilbenes [42], resulting in a cleaner analytical extract phenolic profile. Additionally, three compounds previously detected in the pilot-scale extraction [2]: protocatechuic aldehyde (*m*/*z* 137.0241, −2.02 ppm), kaempferol-3-*O*-glucoside (*m*/*z* 447.0931, −0.49 ppm), and ethyl protocatechuate (*m*/*z* 181.0504, −1.02 ppm), were also recovered and identified in the microencapsulated extract.

Finally, ten compounds were identified only in the IC (GC_PPE_+HP-*β*-CD+MD), and not in the analytical or pilot extracts: five flavonoids, three stilbenes, an adduct of *β*-CD, and a complex of *β*-CD with (*epi*)catechin.

*Flavonoids.* The taxifolin isomer (*m*/*z* 303.0503, −2.32 ppm) showed ions at *m/z* 285.0390, owing to the initial loss of a water molecule and ions at *m*/*z* 177.0184 and 125.0237 due to cleavage of the C ring, respectively. Quercetin (*m*/*z* 301.0354, 0.06 ppm) was identified and confirmed by comparison with a pure standard. Myricetin (*m*/*z* 317.0301, −0.63 ppm), tentatively identified by its fragmentation pattern, produced ions at *m*/*z* 178.9981 (^1,2^A^−^) and 151.0032 (^1,3^A^−^) due to retro-Diels–Alder fragmentation [43], and at *m*/*z* 192.0058 due to the loss of the B ring. Although they were not identified in our previous studies, these compounds have been recovered, detected, and quantified by other authors using microwave-assisted, subcritical water, and conventional extraction techniques [44].

Dihydrokaempferol-*O*-rhamnoside (engeletin) (*m*/*z* 433.1139, −0.38 ppm), a compound previously reported in grape stems [45], was tentatively identified and showed fragment ions at *m*/*z* 269.0446, 287.0550, and 259.0603. An undefined tetrahydroxyisoflavanone (*m*/*z* 287.0561, −0.18 ppm) gave product ions at *m*/*z* 259.0602, 243.0652, and 201.0547, and was provisionally identified as 2,6,7,4′-tetrahydroxyisoflavanone, based on the exact mass and fragmentation pattern. However, as dihydrokaempferol and eriodictyol chalcone have similar structures, the identity of this compound could not be accurately defined using our spectrometric approach.

**Figure 3:**
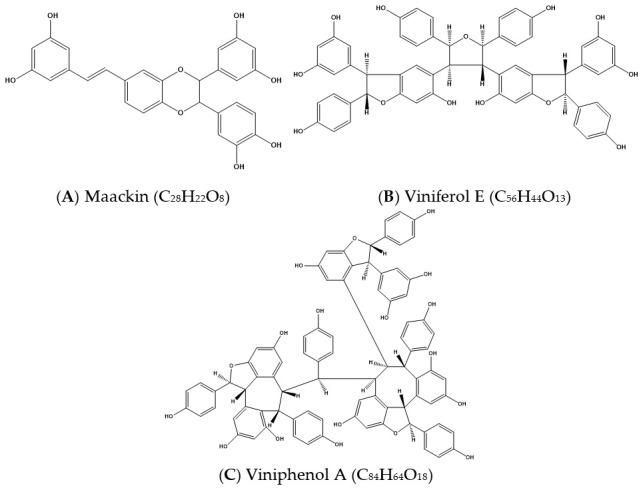
Representative stilbenes tentatively identified in the microencapsulated extract. (**A**) Maackin (C_28_H_22_O_8_); (**B**) Viniferol E (C_56_H_44_O_13_); (**C**) Viniphenol A (C_84_H_64_O_18_).

*Stilbenes*. A stilbenoid dimer (maackin, Figure 3A) (*m*/*z* 485.1242, 0.01 ppm) showed product ions at *m*/*z* 467.1125, 375.0865, and 363.0863, which were generated by the loss of a water molecule (18 Da), resorcinol (110 Da), and 2-hydroxy-4-methylenecyclohexa-2,5-dienone (122 Da), respectively [46]. This compound has a structure consisting of two piceatannol units, and the most likely assignment is maackin A, which was identified previously in *V. vinifera* stalks [47].

A stilbenoid tetramer (Figure 3B) (*m*/*z* 923.2680, 0.68 ppm) was tentatively identified as viniferol E and yielded product ions at *m*/*z* 905.2576, 881.2573, 801.2318, 783.2209 and 707.1898. The product ions at *m*/*z* 905.2576 and 881.2573 were due to a loss of H_2_O (18 Da) and C_2_H_2_O (42 Da), respectively, and at *m*/*z* 801.2318 probably to the loss of the group C_7_H_6_O_2_ (122 Da). The ion at *m*/*z* 801.2318 was further fragmented to ions at *m*/*z* 783.2209 and *m*/*z* 707.1898 by the loss of a water molecule (18 Da) and a phenol group (94 Da), respectively. Viniferol E was previously detected and quantified from grapevine canes by subcritical water extraction [48].

A stilbenoid hexamer, viniphenol A (Figure 3C) (*m*/*z* 679.1969 [M − 2H]^2−^, −1.12 ppm), was detected as a doubly charged ion with product ions at *m*/*z* 905.2584, 585.1543, 491.1126, 453.1333 and 359.0914. The product ion at *m*/*z* 905.2584 shows the presence of a stilbenoid tetramer molecule, probably formed by the loss of a stilbenoid dimer (454 Da) from the deprotonated hexamer. The high-intensity product ion at *m*/*z* 585.1543 could be attributed to the loss of a phenol group (94 Da) from the stilbenoid trimer (*m*/*z* 679). The product ion at *m*/*z* 585.1543 underwent fragmentation to ions at *m*/*z* 491.1126 and *m*/*z* 359.0914, which could be attributed to the loss of a phenol group (94 Da) and a stilbenoid dimer (226 Da), respectively. Finally, a low intensity stilbenoid dimer fragment was observed at *m*/*z* 453.1333. Viniphenol A was previously isolated from *V. vinifera* stalks by centrifugal partition chromatography, while its structure was proposed based on the analysis of spectroscopic data and molecular modeling under NMR conditions [47].

According to the supplier, the HP-*β*-CD used for encapsulation has a maximum *β*-CD impurity of 1.5%. The presence of [*β*-CD+ HCOO]^—^ (*m*/*z* 1179.3679, −0.04 ppm) and a complex of (*epi*)catechin with *β*-CD [*β*-CD +(*epi*)catechin]^—^ (*m*/*z* 1423.5708, −0.71 ppm) were detected and identified by comparison with the mass spectra reported by Żyżelewicz et al. [49]. The detection of this complex by mass spectrometry confirmed the interaction between polyphenols and cyclodextrins, in agreement with the SEM and FTIR analysis.

### 3.5. Encapsulation Efficiency

GC_PPE_ is a complex mixture of various compounds that have different physical and chemical properties and abilities to form interactions and bind within the HP-*β*-CD cavity. As shown earlier, the phenolic compounds in the microencapsulated extract are phenolic acids and derivatives, flavonoids, and stilbenes. The individual EE (%) for twenty of these compounds was calculated and presented in Table 3. The other compounds were identified but not quantified due to their low amounts (see detail Supplementary Materials Table S1).

The average EE for all the analyzed compounds was 80.5 ± 1.1%. Several authors have studied the encapsulation of phenolic compounds from wine byproducts using different encapsulation materials. Davidov-Pardo et al. [30] reported a similar polyphenol EE of 82% for a commercial grape seed extract microencapsulated by spray drying using MD as the wall material. Moschona and Liakopoulou-Kyriakides [50] found a low EE of 55% to 79% for grape marc and lees phenolic extracts from white and red wine encapsulated with alginate and chitosan. Lavelli and Sri Harsha [51] also reported a low EE of 68% in a study where alginate hydrogel was used as an agent to encapsulate phenolic compounds from grape skin. Another study using grape skin extracts, encapsulated in water-in-oil-in-water (W/O/W) double emulsions, observed an improved EE of 87.74 ± 3.12% for anthocyanins [52]. The EE depends on a variety of factors, such as the technique used, the solubility and size of the guest molecule relative to the cavity of the host, the concentrations of the host and guest molecules, the binding constant within the guest and host, etc. [53]. As we did not find a similar study in the literature that employed the same raw material, encapsulant, and analytical methods to determine the concentration of guest molecules, we also analyzed the individual encapsulation of twenty compounds present in the IC (GC_PPE_+HP-*β*-CD+MD).

The average EE for the phenolic acids and derivatives was 81.5 ± 0.7%, the lowest value being obtained for protocatechuic acid-*O*-hexoside 1 (54.3 ± 2.4%) and the highest for hydroxybenzaldehyde (95.8 ± 0.6%). The EE for protocatechuic acid was 66.8 ± 9.8%, higher than the value reported by Taofiq et al. [54], who determined an EE of 50.3% for protocatechuic acid encapsulated by the atomization/coagulation technique, using sodium alginate in combination with calcium chloride (CaCl_2_) to promote alginate gelation. The EE for hydroxybenzaldehyde obtained here is higher than the value (46.50%) reported for *p*-hydroxybenzaldehyde in a *β*-CD inclusion complex [55].

The EE for gallic acid, 83.4 ± 0.6%, was slightly higher than the value reported by da Rosa et al. [56], who determined an EE of 80.0 ± 1.4% for microencapsulated gallic acid using *β*-CD and the lyophilization method. However, Olga et al. [53] obtained a higher EE of 89.22% in a complex with HP-*β*-CD, a result that was reduced to 77.34% when the gallic acid was co-encapsulated with *trans*-ferulic acid. The authors suggested a possible antagonistic relationship between the two phenols in the HP-*β*-CD cavity.

The EE of 87.6 ± 0.5% observed for ellagic acid pentoside was considerably higher than the 55.2% reported for ellagic acid (aglycone) encapsulated with polyvinyl alcohol [57]. The EE for caftaric acid—a hydroxycinnamic acid—was very high (87.1 ± 2.2%) in comparison with the values reported for other hydroxycinnamic acids, such as the essential oil-encapsulated chitosan-*ρ*-coumaric acid (42 ± 1%) [58] and chlorogenic acid (77.5%) in *β*-CD nanosponges [59], respectively. Finally, to our knowledge, this is the first time that the EE for ethyl protocatechuate (69.7 ± 5.5%) and protocatechuic aldehyde (75.6 ± 4.2%) has been reported.

The average EE for flavonoids was 85.2 ± 2.4%. The values for astilbin isomer (2) and astilbin (1) were particularly high, 92.0 ± 1.1% and 82.7 ± 2.3%, respectively. Zheng and Zhang [60] reported a lower EE of 80.1% for astilbin encapsulated with zein−caseinate nanoparticles by the antisolvent method.

Quercetin-3-*O*-glucuronide (88.0 ± 3.1%) and quercetin-*O*-glucoside (81.8 ± 7.0) also had high EE values. Tchabo et al. [61] obtained a lower EE (63.90–66.45%) for quercetin- 3-*O*-glucoside in a spray-dried mulberry leaf extract prepared with MD, yet the EE was higher (91.71–93.95%) when sodium carboxymethyl cellulose was used. To the best of our knowledge, no EE values have been previously reported for quercetin-3-*O*-glucuronide.

The EE for eriodictyol was 74.2 ± 4.7%, similar to the almost 70% reported for naringenin (another flavanone) in polymer PLGA nanoparticles prepared by an emulsion-diffusion-evaporation method [62].

The stilbene group had the lowest mean EE (78.6 ± 1.9%), mainly because of the low value obtained for (*E*)-resveratrol (32.7 ± 2.8%). Nevertheless, previous studies have shown that the cyclodextrin encapsulation of resveratrol increases its solubility, stability, and bioactivity (antioxidant and anticarcinogenic properties) [15]. Furthermore, the HP-*β*-CD complex exhibits a strong H-bonding interaction with molecules such as oxyresveratrol [63]. In our study, we found a high EE (97.0 ± 0.6%) for restrytisol (A or B), an oxidized resveratrol dimer, which may be related to the van der Waals force interaction and hydrogen bonding between the guest compound and the HP-*β*-CD.

A good EE was obtained for stilbene dimers such as pallidol (74.6 ± 3.8%), (*E*)-ε-viniferin (76.8 ± 3.5%), and an undefined resveratrol dimer (94.1 ± 2.1%). Previously, ε-viniferin, a resveratrol dimer, was encapsulated in phospholipid-based multi-lamellar liposomes called spherulites or onions. This formulation gave a lower EE (58 ± 3%) than in our study, but increased the water solubility of this stilbene more than five-fold and provided protection against its UV-induced isomerization [64]. Finally, the low EE (65.8 ± 8.6%) obtained for the stilbenoid tetramer is probably due to its large size and polar surface area.

### 3.6. Antioxidant Capacity

Several authors suggest that the antioxidant capacity of phenolic compounds is improved by encapsulation in cyclodextrins [15]. The ORAC value has been applied to standardize the antioxidant activity of herbal extracts and foods, and is widely used as an accurate indicator of antioxidant activity in vivo [65].

The antioxidant capacity of the IC (GC_PPE_+HP-*β*-CD+MD) by ORAC-FL was 5300 ± 472 µmol TE/g DW, similar to the 4612 ± 155 µmol TE/g DW reported in our previous GC_PPE_ study [2]. The GC_PPE_ microencapsulated with HP-*β*-CD retains its antioxidant capacity and its formulation may improve stability, solubility, and bioavailability for applications in the food, cosmetic and pharmaceutical industries.

## 4. Conclusions

Phenolic compounds from a grape cane pilot-plant extract were successfully encapsulated in an inclusion complex (GC_PPE_+HP-*β*-CD+MD). The microencapsulated extract was rich in stilbenes, especially oligomers, flavonoids, and phenolic acids. A complex of (*epi*)catechin and *β*-CD was detected by mass spectrometry, which confirmed the interaction between polyphenols and cyclodextrin. The formation of the inclusion complex was also supported by FTIR-ATR and SEM analyses. HP-*β*-CD provided a high EE for phenolic compounds, with a mean of 80.5 ± 1.1%, the highest values being obtained for restrytisol (97.0 ± 0.6%), stilbenoid heterodimer (1) (96.8 ± 0.4%) and hydroxybenzaldehyde (95.8 ± 0.6%), and the lowest for (*E*)-resveratrol (32.7 ± 2.8%). The antioxidant capacity of the inclusion complex was similar to the unencapsulated extract. Considering the protection afforded the phenolic compounds by the inclusion complex, it is expected that the formulation may improve their stability, solubility, and bioavailability in water-soluble applications for the food and pharmaceutical industries.

## 5. Patents

The preparation of grape canes before extraction in the pilot plant followed the procedure described in the Patent [13]. The formulation of the microparticles of phenolic compounds from the extract of grape canes of *V. vinifera* and the application of HP-*β*-CD to form the IC and the use of MD as a coating agent are described in the Patent [17].

## Figures and Tables

**Table 1:** Physical characteristics and process parameters for the grape cane phenolic extract (GC_PPE_) and inclusion complex (GC_PPE_+HP-β-CD+MD).

	**Process Parameters**			**Size Distribution**		**Property of Powders**	
	**Moisture (%)**	**Solids** **(g)**	**PY** **(%)**	**D50** **(µm)**	***Span***	**Bulk Density (g/mL)**	**Angle of Repose** **(°)**
GC_PPE_	6.3 ± 1.5 ^a^	11.0 ± 0.1 ^a^	38.4 ± 1.2 ^a^	17.5 ± 0.4 ^a^	6.15 ± 0.1 ^a^	0.10 ± 0.01 ^a^	34.8 ± 0.5 ^a^
IC (GC_PPE_+HP-*β*-CD+MD)	7.2 ± 0.3 ^a^	28.9 ± 0.1 ^b^	83.8 ± 2.6 ^b^	10.9 ± 0.9 ^b^	6.14 ± 0.1 ^a^	0.19 ± 0.01 ^b^	36.9 ± 1.3 ^a^

Results are expressed as means ± standard deviations, and values with different superscripts letters in a column indicate significant differences at *p* < 0.05.

**Table 2:** Identification of phenolic compounds in the IC (GC_PPE_+HP-*β*-CD+MD), an adduct of *β*-CD and a complex between *β*-CD with (*epi*)catechin using LC-ESI-LTQ-Orbitrap-MS in negative mode.

Compounds	t_R_ (min)	Accurate Mass [M − H]^−^	Theo. Mass	Error (ppm)	MS/MS Ions (Intensity)	Molecular Formula
Gallic acid *	4.33	169.0138	169.0142	−2.41	125.0236(100)	C_7_H_6_O_5_
Monogalloyl-glucose	5.79	331.0662	331.0671	−2.65	169.0134(100), 125.0238(5)	C_13_H_16_O_10_
[*β*-CD+HCOO]^−^	7.21	1179.3679	1179.3680	−0.04	675.8472(100), 797.1872(80), 332.8267(80), 734.6646(80)	C_43_H_72_O_37_
Protocatechuic acid-*O*-hexoside (1)	7.35	315.0718	315.0722	−1.07	153.0186(100), 109.0288(10)	C_13_H_16_O_9_
Protocatechuic acid	7.55	153.0190	153.0193	−2.16	109.0288(100)	C_7_H_6_O_4_
Protocatechuic acid-*O*-hexoside (2)	8.46	315.0717	315.0722	−1.30	153.0189(100), 109.0290(10)	C_13_H_16_O_9_
Syringic acid hexoside	8.66	359.0933	359.0925	2.21	197.0445(100)	C_15_H_20_O_10_
Protocatechuic aldehyde	9.32	137.0241	137.0244	−2.02	93.0338(100), 109.0285(70)	C_7_H_6_O_3_
Procyanidin dimer (1)	10.34	577.1348	577.1351	−0.69	425.0864(100), 407.0760(70), 451.1019(45), 289.0706(35)	C_30_H_26_O_12_
Procyanidin dimer (2)	10.77	577.1346	577.1351	−0.35	425.0861(100), 407.0757(60), 451.1017(35), 289.0705(25)	C_30_H_26_O_12_
Catechin *	11.29	289.0722	289.0718	1.43	245.0817(100), 205.0504(40), 179.0348(20)	C_15_H_14_O_6_
Complex [*β*-CD+(*epi*)catechin]^—^	13.00	1423.5708	1423.5718	−0.71	1245.4839(100), 1303.5255(85), 1101.4424(60), 1365.5260(60), 1.083.4313(60)	C_57_H_84_O_41_
Epicatechin *	13.29	289.0715	289.0718	−1.00	245.0812(100), 205.0499(40), 179.0343(15)	C_15_H_14_O_6_
Restrytisol (A or B)	15.08	471.1441	471.1449	−1.86	255.0653(100), 377.1017(65), 349.1068(45)	C_28_H_24_O_7_
Epicatechin gallate *	16.86	441.0821	441.0827	−1.30	289.0705(100), 169.0136(30)	C_22_H_18_O_10_
Taxifolin *	17.00	303.0508	303.0510	−0.78	285.0397(100), 177.0186(15), 125.0238(10)	C_15_H_12_O_7_
Astilbin (1)	17.36	449.1084	449.1089	−1.12	303.0495(100), 285.0392(85), 151.0028(30)	C_21_H_22_O_11_
Taxifolin isomer	17.43	303.0503	303.0510	−2.32	285.0390(100), 177.0184(15), 125.0237(10)	C_15_H_12_O_7_
Stilbenoid heterodimer (caraphenol B/C)	17.78	469.1292	469.1293	−0.06	451.1189(100), 363.0875(35), 375.0872(30), 281.0452(2)	C_28_H_22_O_7_
(*E*)-Piceatannol *	17.95	243.0665	243.0663	0.79	225.0551(100), 201.0552(75), 159.0447(20)	C_14_H_12_O_4_
Kaempferol-3-*O*-glucoside *	18.39	447.0931	447.0933	−0.49	284.0315(100), 285.0392(75), 327.0497(15), 255.0286(10)	C_21_H_20_O_11_
Ethyl protocatechuate	18.49	181.0504	181.0506	−1.02	153.0187(100), 152.0110(15), 109.0289(5)	C_9_H_10_O_4_
Dihydrokaempferol-*O*-rhamnoside	18.77	433.1139	433.1140	−0.38	269.0446(100), 287.0550(40), 259.0603(15)	C_21_H_22_O_10_
Eriodictyol-*O*-glucoside	19.01	449.1089	449.1089	−0.04	287.0552(100), 151.0031(5)	C_21_H_22_O_11_
Stilbenoid dimer	19.16	469.1292	469.1293	−0.07	363.0857(100), 375.0857(20), 451.1168(5)	C_28_H_22_O_7_
Undefined (tetrahydroxyisoflavanone)	19.18	287.0561	287.0561	−0.18	259.0602(100), 243.0652(20), 201.0547(5)	C_15_H_12_O_6_
Viniferin diglycoside	19.25	777.2397	777.2400	−0.36	615.1854(100), 453.1330(80)	C_40_H_42_O_16_
Myricetin	19.39	317.0301	317.0303	−0.63	178.9981(100), 151.0032(45), 192.0058(10)	C_15_H_10_O_8_
Pallidol	19.50	453.1349	453.1344	2.24	359.0922(100), 265.0499(10)	C_28_H_22_O_6_
(*E*)-resveratrol *	20.23	227.0715	227.0714	0.77	185.0607(100), 183.0816(35), 159.0814(30)	C_14_H_12_O_3_
Stilbenoid dimer (resveratrol dimer)	20.56	453.1351	453.1344	1.57	359.0916(100), 289.0861(5)	C_28_H_22_O_6_
Stilbenoid dimer (maackin)	20.62	485.1242	485.1242	0.01	375.0865(100), 467.1125(15), 363.0863(10)	C_28_H_22_O_8_
Resveratrol-*O*-hexoside	20.79	615.1868	615.1872	−0.62	453.1334(100)	C_34_H_32_O_11_
Eriodictyol *	20.97	287.0558	287.0561	−0.92	151.0031(100), 135.0446(5)	C_15_H_12_O_6_
Stilbenoid tetramer (viniferol E)	21.11	923.2680	923.2674	0.68	905.2576(100), 707.1898(55), 801.2318(50), 881.2573(20), 783.2209(10)	C_56_H_44_O_13_
Stilbenoid hexamer (viniphenol A)	21.12	679.1969[M − 2H]^2−^	679.1974[M − 2H]^2−^	−1.12	585.1543(100), 491.1126(10), 359.0914(5), 905.2584(2.5), 453.1333(2)	C_84_H_64_O_18_
Quercetin *	21.16	301.0354	301.0354	0.06	178.9987(100), 151.0038(80)	C_15_H_10_O_7_
Hopeaphenol *	21.34	905.2607	905.2627	−2.24	811.2152(100), 717.1741(65), 451.1173(10)	C_56_H_42_O_12_
Isohopeaphenol *	21.47	905.2588	905.2568	2.15	811.2178(100), 717.1752(40)	C_56_H_42_O_12_
(*E*)-ε-viniferin *	21.59	453.1348	453.1344	1.03	359.0928(100), 347.0923(50), 435.1238(25)	C_28_H_22_O_6_
(*E*)-ω-viniferin	21.72	453.1346	453.1344	0.62	359.0927(100), 347.0925(50), 435.1234(30), 411.1233(20)	C_28_H_22_O_6_
Stilbenoid tetramer (vitisin A/B/C/D)	21.90	905.2599	905.2627	−3.05	799.2157(100), 887.2482(70), 811.2158(50), 359.0913(35), 545.1599(15)	C_56_H_42_O_12_

* Compounds identified via matching with authentic standards. t_R_, retention times. Isomers are presented in brackets. Stilbenoid hexamer appeared as a doubly charged ion.

**Table 3:** Encapsulation efficiency (%).

Compounds	EE (%)
**Phenolic Acids and Derivatives**	
Protocatechuic acid-*O*-hexoside (1)	54.3 ± 2.4
Protocatechuic acid	66.8 ± 9.8
Ethyl protocatechuate	69.7 ± 5.5
Protocatechuic aldehyde	75.6 ± 4.2
Gallic acid	83.4 ± 0.6
Caftaric acid	87.1 ± 2.2
Ellagic acid pentoside	87.6 ± 0.5
Hydroxybenzaldehyde	95.8 ± 0.6
**Weighted Average**	**81.5 ± 0.7**
**Flavonoids**	
Eriodictyol	74.2 ± 4.7
Quercetin-*O*-glucoside	81.8 ± 7.0
Quercetin-3-*O*-glucuronide	88.0 ± 3.1
Astilbin (1)	82.7 ± 2.3
Astilbin (2)	92.0 ± 1.1
**Weighted Average**	**85.2 ± 2.4**
**Stilbenes**	
(*E*)-resveratrol	32.7 ± 2.8
Stilbenoid tetramer (hopeaphenol/isohopeaphenol)	65.8 ± 8.6
Pallidol	74.6 ± 3.8
(*E*)-ε-viniferin	76.8 ± 3.5
Stilbene dimer (resveratrol dimer)	94.1 ± 2.1
Stilbenoid heterodimer (caraphenol B/C)	96.8 ± 0.4
Restrytisol (A or B)	97.0 ± 0.6
**Weighted Average**	**78.6 ± 1.9**
**Total Weighted Average**	**80.5 ± 1.1**

Results are given as means ± standard deviations. For each phenolic class, we calculated the weighted average of the concentration of the respective metabolites. The total weighted average was calculated by weighting all quantified compounds. All encapsulation efficiencies were calculated according to Equation (4) (Section 2.8).

## Data Availability

Data is contained within the article and Supplementary Materials.

## Supplementary Materials

The following are available online at https://www.mdpi.com/article/10.3390/antiox10071130/s1, Table S1: Identification and quantification of phenolic compounds from GC_PPE_ using LC-ESI-LTQ-Orbitrap-MS in negative mode.
