# Supplementary material for: Encapsulation of Phenolic Compounds from a Grape Cane Pilot-Plant Extract in Hydroxypropyl Beta-Cyclodextrin and Maltodextrin by Spray Drying
Source: Antioxidants (Basel). 2021 Jul 15;10(7):1130. doi: 10.3390/antiox10071130 (PMC8301162; doi:10.3390/antiox10071130)
Supplement: Supplementary file 1 [file antioxidants-10-01130-s001.zip › antioxidants-1275960-supplementary.pdf]

antioxidants

MDPI

Supplementary Data

Encapsulation of Phenolic Compounds from a Grape Cane Pilot-Plant Extract in Hydroxypropyl Beta-Cyclodextrin and Maltodextrin by Spray Drying

Danilo Escobar-Avello [1,2], Javier Avendaño-Godoy [3], Jorge Santos [4,5], Julián Lozano-Castellón [1,6], Claudia Mardones [7], Dietrich von Baer [7], Javiana Luengo [3], Rosa M. Lamuela-Raventós [1,6], Anna Vallverdú-Queralt [1,6,*] and Carolina Gómez-Gaete [2,3,*]

1 Department of Nutrition, Food Science and Gastronomy XaRTA, Faculty of Pharmacy and Food Sciences Institute of Nutrition and Food Safety (INSA-UB), University of Barcelona, 08028 Barcelona, Spain; daniescobar01@ub.edu (D.E.-A.); julian.lozano@ub.edu (J.L.-C.); lamuela@ub.edu (R.M.L.-R.)
2 Unidad de Desarrollo Tecnológico, Universidad de Concepción, 4191996 Coronel, Chile
3 Departamento de Farmacia, Facultad de Farmacia, Universidad de Concepción, 4191996 Concepción, Chile; jaavendano@udec.cl (J.A.-G.); jluengo@udec.cl (J.L.)
4 DEMad, Instituto Politécnico de Viseu, 3504-510 Viseu, Portugal; jsantosu@estgv.ipv.pt (J.S.)
5 LEPABE—Faculty of Engineering, University of Porto, 4200-465 Porto, Portugal

Table S1: Identification and quantification of phenolic compounds from GC_{PPE} using LC-ESI-LTQ-Orbitrap-MS in negative mode.

Compounds	tR (min)	Accurate mass $[M-H]^-$	mg/kg DW
**PHENOLIC ACIDS AND ALDEHYDES**			
Monogalloyl-glucose	3.63	331.0668	42 ± 6
Gallic acid*	4.29	169.0141	279 ± 58
Protocatechuic acid-*O*-hexoside (1)	7.39	315.0719	388 ± 53
Protocatechuic acid	7.67	153.0192	266 ± 47
Protocatechuic acid-*O*-hexoside (2)	8.52	315.0718	NQ
Syringic acid hexoside	8.76	359.0981	NQ
Caftaric acid	9.27	311.0406	135 ± 21
Protocatechuic aldehyde	9.47	137.0242	711 ± 100
Hydroxybenzoyl hexoside	9.95	299.0770	59 ± 10
4-Hydroxybenzoic acid*	10.04	137.0243	69 ± 8
Coutaric acid	11.17	295.0457	97 ± 20
Hydroxybenzaldehyde	11.75	121.0294	1295 ± 131
Ellagic acid hexoside	14.00	463.0518	NQ
Gallic acid ethyl ester*	14.30	197.0453	45 ± 8
Ellagic acid pentoside	16.03	433.0410	164 ± 16
Ellagic acid*	16.95	300.9986	284 ± 7
Ethyl protocatechuate	18.58	181.0504	283 ± 45
**FLAVONOIDS**			
Flavanols			
Catechin*	11.40	289.0715	249 ± 38
Epicatechin*	13.36	289.0714	NQ
Procyanidin A-type dimer	15.80	575.1194	NQ
Flavanones			
Eriodictyol-*O*-glucoside (1)	13.58	449.1090	NQ
Eriodictyol-*O*-glucoside (2)	19.05	449.1089	NQ
Eriodictyol*	21.07	287.0555	143 ± 34
Flavanonols			
Taxifolin*	17.03	303.0506	269 ± 52
Astilbin (1)	17.39	449.1090	1352 ± 208
Astilbin (2)	18.23	449.1084	479 ± 76
Flavonols			
Quercetin-*O*-glucoside*	17.21	463.0879	43 ± 7
Quercetin-3-*O*-glucuronide*	17.28	477.0670	85 ± 13
Kaempferol-3-*O*-glucoside*	18.41	447.0930	NQ
**STILBENES**			
Resveratrol C-hexoside	13.68	389.1238	NQ
Restrytisol (A or B)	15.12	471.1442	421 ± 65
Oxyresveratrol	15.51	243.0659	NQ

Oxidized stilbenoid dimer (1)	16.19	471.1449	NQ
Stilbenoid dimer 1 (Caraphenol B/C)	17.81	469.1286	1683 ± 236
Oxidized stilbenoid dimer (2)	18.90	471.1446	NQ
Stilbenoid dimer 2 (heterodimer)	19.26	469.1288	NQ
Pallidol	19.52	453.1341	393 ± 71
(*E*)-resveratrol*	20.28	227.0709	502 ± 50
Stilbene dimer (resveratrol dimer)	20.63	453.1338	239 ± 51
Stilbenoid trimer	20.77	681.2123	NQ
Resveratrol dimer-*O*-hexoside	20.85	615.1866	NQ
Stilbenoid dimer 3 (Scirpusin A)	21.31	469.1289	NQ
Stilbenoid tetramer (Hopeaphenol/Isohopeaphenol)	21.44	905.2580	974 ± 227
(*E*)-ε-viniferin*	21.69	453.1336	3962 ± 485

Data previously published by Escobar-Avello et al [2]. (*) Identification by comparison with a standard. tR., retention time. Isomers are shown in parentheses. Quantification of phenolic compounds in a grape cane pilot plant extract (expressed in mg/kg DW). NQ means identified, but not quantified, due to their low levels (between LOD and LOQ). Results are expressed as means ± standard deviations.
